# Dietary approaches to support cognition in older adults: a systematic review

**DOI:** 10.3389/fnut.2026.1869091

**Published:** 2026-07-09

**Authors:** Britton Woolsey, Keya Sen

**Affiliations:** School of Health Administration, College of Health Professions, Texas State University, San Marcos, TX, United States

**Keywords:** alzheimer's disease, dementia, dietary interventions, healthy aging, personalized nutrition

## Abstract

**Background:**

Older adults, particularly those residing in long-term care, experience disproportionate rates of cognitive decline and Alzheimer's disease (AD). While isolated nutrient supplementation has demonstrated limited clinical efficacy, comprehensive whole-food dietary patterns may offer significant neuroprotective benefits through complex nutrient synergy. This systematic review evaluates the efficacy of the Mediterranean, Nordic, Okinawan, and plant-based dietary approaches in mitigating cognitive decline and reducing dementia risk in older populations.

**Methods:**

The study protocol was prospectively registered with the International Prospective Register of Systematic Reviews (PROSPERO) under the registration ID CRD420261349593. Conducted in accordance with PRISMA 2020 guidelines, a systematic search of PubMed, Web of Science, CINAHL, and ScienceDirect was performed to identify peer-reviewed articles published between January 2021 and the present. Eligible studies included randomized controlled trials (RCT), prospective cohort studies, and longitudinal studies evaluating the impact of whole-food dietary patterns on cognitive outcomes in adults aged 60 and older.

**Results:**

Out of 622 initial records, 16 articles met all inclusion criteria. The synthesized evidence demonstrates that high adherence to these comprehensive dietary patterns is consistently associated with improved memory, enhanced executive function, and a reduced incidence of AD. These cognitive improvements are driven by interconnected physiological mechanisms, including reduced systemic inflammation, improved vascular integrity, favorable shifts in the gut microbiome, and optimized circulating endocannabinoid profiles. Additionally, the magnitude of these benefits is frequently modulated by individual biological factors, such as sex and APOE genotype.

**Conclusion:**

Whole-food dietary patterns provide an effective, evidence-based framework for preserving cognitive resilience compared to single-nutrient interventions. Integrating these nutrient-dense diets into public health initiatives and long-term care settings offers a powerful strategy for neuroprotection, highlighting the need to advance personalized nutrition strategies in future clinical trials.

## Graphical Abstract

**Figure:**
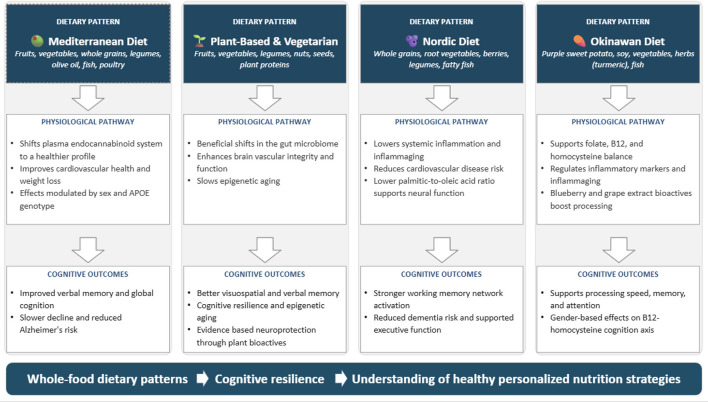

## 1 Introduction

Older adults in long-term care (LTC) experience disproportionately high rates of chronic disease, frailty, and malnutrition, all of which contribute to accelerated cognitive decline (1). Dementia affects more than 57 million people worldwide, and AD accounts for 60 to 80% of these cases (2). Within institutional settings, the prevalence and severity of AD and related dementias are further exacerbated by “inflamm-aging,” a state of chronic and low-grade inflammation that promotes neurodegeneration (3, 4). Nutritional status, commonly assessed through the Mini Nutritional Assessment (MNA), is a critical indicator of vulnerability; lower MNA scores are consistently associated with more advanced dementia staging and greater cognitive impairment (5).

The urgency of this research is driven by the rapid global rise in AD, now the leading cause of disability among older adults in long term care (1, 2). Although age remains the strongest risk factor, the disproportionate rates of cognitive impairment in long term care are intensified by nutritional inadequacy, a condition in which residents meet caloric needs but lack the bioactive compounds required to maintain neural integrity (6). The documented limitations of the supplement-first model in clinical practice further justify this review. High dose vitamin supplementation has repeatedly failed to reproduce the cognitive protection observed in whole food dietary cohorts (7, 8). This gap underscores the importance of the food matrix and nutrient synergy, including interactions between vegetable-based fats, fiber, and polyphenols that reduce neuroinflammation more effectively than isolated nutrients (9, 10). Comprehensive dietary modifications can induce beneficial metabolic shifts; for instance, ketogenic approaches like the modified Atkins diet have been shown to provide alternative energy sources for the brain, potentially improving memory in older adults with mild cognitive impairment due to AD (11).

Identifying dietary patterns that enhance healthspan rather than simply prolong lifespan offers a practical and scalable strategy for long term care settings. Such approaches have the potential to reduce the economic burden of advanced dementia while improving functional independence and overall quality of life for residents (12). Therefore, the primary objective of this systematic review is to evaluate the clinical efficacy of comprehensive, whole-food dietary patterns in mitigating cognitive decline and reducing the risk of AD and related dementias in older adults, with a specific focus on the Mediterranean, Nordic, Okinawan, and plant-based approaches.

## 2 Methods

### 2.1 Eligibility criteria

To ensure the synthesis of high-quality clinical data, specific inclusion and exclusion criteria were established prior to the search. These parameters are structured using the PICOS (Population, Intervention, Comparison, Outcome, Study type) framework, as detailed in Table 1.

**Table 1:** PICOS eligibility criteria.

Criteria	Included	Excluded
Population	Older adults aged 60 and older, residing in community or long-term care settings.	Non-human studies, populations under age 60.
Intervention	Comprehensive whole-dietary patterns (Mediterranean, Nordic, Okinawan, plant-based/vegetarian).	Single nutrient or isolated supplement interventions.
Comparison	Any relevant diet or standard care.	Not applicable.
Outcomes	Quantitative measures of cognitive performance, executive function, clinical markers of neurodegeneration, dementia, and Alzheimer's disease.	Studies not assessing cognitive performance or related biomarkers.
Study type	RCT, prospective cohort studies, and longitudinal studies published in english.	Meta-analyses, previous systematic reviews, and articles not published in english.

### 2.2 Search strategy

This systematic review was conducted in accordance with the Preferred Reporting Items for Systematic Reviews and Meta-Analyses (PRISMA) 2020 guidelines. The study protocol was prospectively registered with the International Prospective Register of Systematic Reviews (PROSPERO) under the registration ID CRD420261349593. This registration ensured transparency and minimized the risk of duplication or bias in the selection process.

Peer-reviewed studies were identified through comprehensive and structured searches of four primary electronic databases: PubMed, Web of Science, CINAHL, and ScienceDirect. To capture the most current clinical evidence, search limits were restricted to articles published in the English language between January 1, 2021, and the present, ensuring the synthesis of studies reflecting the most current clinical standards and modern dietary habits. Because nutritional science and the evaluation of the food matrix have evolved rapidly in recent years, this contemporary window provided a highly robust dataset of 622 initial records. This approach ensured the final analysis was based on the highest-quality, recent randomized controlled trials and cohort studies, accurately reflecting modern whole-food interventions. The search strategy utilized a combination of Medical Subject Headings (MeSH) terms and Boolean operators to link dietary interventions with cognitive outcomes in aging populations. Specifically, the search terms were organized into three main concepts: dietary interventions (using keywords such as “Mediterranean diet” OR “Nordic diet” OR “Okinawa diet” OR “plant-based diet” OR “vegetarian diet” OR “vegan diet” OR “intermittent fasting” OR “caloric restriction”), targeted cognitive outcomes (using terms such as “cognitive decline” OR “dementia” OR “Alzheimer^*^ disease”), and the intended demographic (restricted by using terms such as “older adults” OR “healthy aging”).

### 2.3 Study selection and data extraction

**Figure 1:**
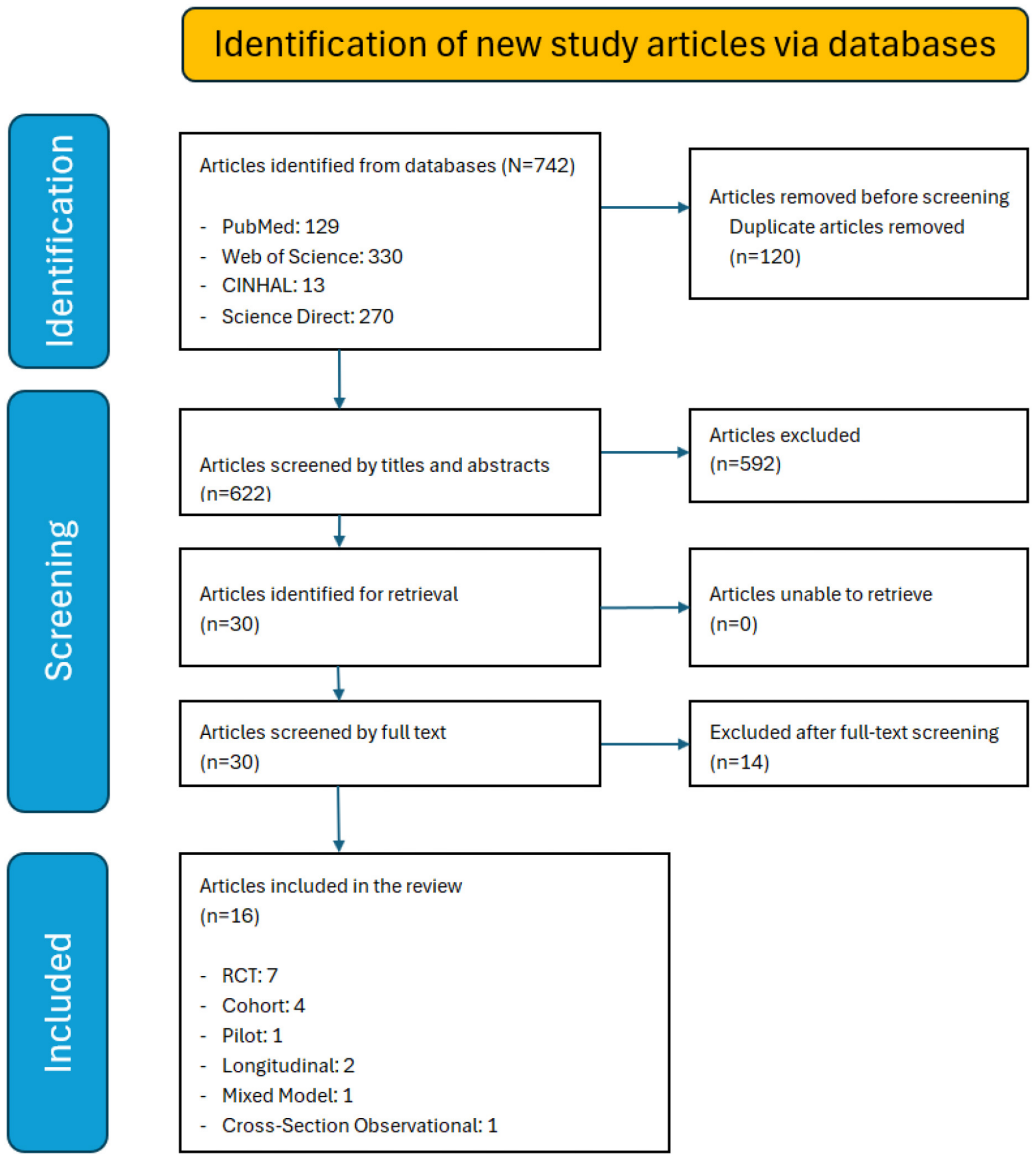
PRISMA flow diagram.

The systematic search and screening process is detailed in the PRISMA 2020 flow diagram, Figure 1. The initial search yielded 622 unique records, from which 30 full-text articles were reviewed for eligibility. During this final full text screening phase, 14 articles were excluded because they failed to meet the specific eligibility criteria. A PRISMA flow diagram was utilized to document the rigorous identification, screening, and selection process. The 16 included studies were synthesized qualitatively to compare the efficacy of the major dietary patterns in mitigating the risks of AD and related dementias.

The 16 included studies were evaluated for methodological quality and risk of bias. The selected literature, comprising RCT, prospective cohort studies, and cross-sectional analyses, generally demonstrated high quality. Assessments confirmed robust dietary tracking methods, standardized cognitive testing, and adequate control for confounding variables such as age, sex, baseline metabolic health, and genetic risk factors. Data extraction from these studies focused on four key pillars: study design and quality, population characteristics, dietary pattern assessment methodology, and specific cognitive outcomes and biomarkers, such as plasma endocannabinoids (13), homocysteine levels (14), and inflammatory cytokines (15).

### 2.4 Quality assessment and synthesis

Due to the significant heterogeneity across the 16 included articles in terms of study design, intervention duration, and cognitive assessment tools, a quantitative meta-analysis was not feasible. Instead, the data was synthesized qualitatively to compare the efficacy of the major dietary patterns in mitigating the risks of Alzheimer's disease and related dementias. To unify the interpretation of these different levels of evidence, the synthesis framework prioritized findings from randomized controlled trials to establish clinical efficacy and short-term cognitive impacts. Data from prospective cohort studies and cross-sectional observational studies were also integrated to provide critical, long-term context regarding real-world dietary adherence and the gradual progression of cognitive decline. Finally, pilot studies were evaluated primarily for their mechanistic insights and feasibility, rather than definitive clinical outcomes. Throughout this qualitative synthesis, study quality and the risk of bias were systematically assessed, ensuring that the final clinical recommendations were driven by the highest-quality, evidence available.

## 3 Results

Across the 16 included studies, researchers evaluated the clinical efficacy of comprehensive, whole-food dietary patterns in older adults. The specific cognitive impacts, physiological pathways, and key foods associated with each evaluated dietary pattern are outlined in Table 2. The detailed study designs, population characteristics, and baseline metrics for all included articles are summarized in Table 3.

**Table 2:** Dietary patterns, key foods, and physiological outcomes.

Dietary pattern	Key foods	Cognitive impact	Physiological pathways	References
Mediterranean	Fruits, vegetables, whole grains, legumes, olive oil, fish, poultry.	Improved verbal memory; better global cognition and slower decline.	Improved cardiovascular health and weight loss; shifts plasma endocannabinoids to a healthier profile.	Soldevila-Domenech et al. (13), Soldevila-Domenech et al. (18), Chou et al. (16), Jennings et al. (17), Mamalaki et al. (19)
Vegetarian/Vegan	Fruits, vegetables, legumes, nuts, seeds, plant-based proteins.	Better visuospatial and verbal memory; slower cognitive decline.	Beneficial gut microbiota changes and enhanced brain vascular function.	Nijssen et al. (21), Ni et al. (22), Ni et al. (27), Wu et al. (10)
Nordic	Whole grains, root vegetables, berries, legumes, fatty fish.	Stronger working memory network activation; reduced dementia risk.	Lower systemic inflammation and reduced cardiovascular disease risks.	Kien and Dumas (23), Buchholz et al. (11), Norgren et al. (24)
Okinawa	Purple sweet potatoes, vegetables, soy, herbs (including turmeric), fish.	Supports processing speed, memory, and attention.	Supports B12, folate, and homocysteine levels and regulates inflammatory markers.	Ting et al. (14), O'Donoghue et al. (15), Cheatham et al. (25), Amone et al. (26)

**Table 3:** Comprehensive summary of included studies and effect sizes.

References	Study design	Population	Sample size	Duration	Diet type/ intervention	Cognitive domains measured	Cognitive assessment tool	Key findings	Effect size (effectiveness)	Association result
Soldevila-Domenech et al. (13)	Prospective cohort	Older adults with metabolic syndrome	102	3 years	Mediterranean Diet (Endocannabinoids interaction)	Memory, executive functions, global cognition	Neuropsychological test battery	Men experienced greater memory improvements than women; endocannabinoid ratios were associated with cognitive changes, modulated by APOE genotype.	OEA/AEA ratio linked to memory improvements (beta = 0.66).	Positive (Modulated by sex/APOE)
Soldevila-Domenech et al. (18)	Longitudinal	Adults with overweight/ obesity, metabolic syndrome, normal cognition	487	1 and 3 years	Energy-restricted Mediterranean Diet	Memory, executive functions, global cognition, attention, inhibition	Comprehensive neurocognitive test battery	The Mediterranean diet prevented age-related cognitive decline, with memory and executive functions improving. Higher adherence linked to greater memory improvement.	Higher adherence associated with memory improvement (beta = 0.13).	Positive
Chou et al. (16)	Cluster-RCT	Community-dwelling older adults in Taiwan	80	8 weeks	Mini-flipped, game-based Mediterranean diet learning program	Global cognitive function, subjective cognitive dysfunction	MoCA, cognitive failures questionnaire (CFQ)	The experimental group exhibited significantly improved Mediterranean diet behavior and global cognitive function.	Post-test MoCA significantly higher in experimental group (*p* = 0.017).	Positive
Jennings et al. (17)	RCT	Older adults at risk of dementia	86	24 weeks (plus 48 weeks follow-up)	Mediterranean diet alone or with physical activity	General cognition, processing speed, executive function, memory	Trail making test, controlled oral word association, wechsler memory, rey auditory verbal learning	Intervention effectively improved general cognition and verbal memory over 24 weeks, but improvements were not maintained at 48 weeks.	General cognition (T3-T1) difference 0.29.	Positive (short-term)
Mamalaki et al. (19)	Longitudinal	Participants with normal cognition at baseline	537	Mean follow-up 2.9 years	Mediterranean diet (MedDietScore)	Alzheimer's disease (AD) incidence	Global cognition score, clinical evaluation	Low adherence to the Mediterranean diet significantly increased AD risk specifically in individuals with low genetic risk.	HR = 10.416 for low MedDiet adherence in low genetic risk group.	Positive
Nijssen et al. (21)	RCT	Healthy older adults	28	16-week intervention	Mixed nuts	Visuospatial memory, verbal memory, executive function, psychomotor speed	CANTAB	Mixed nut consumption beneficially affected brain vascular function and improved visuospatial and verbal memory, but not executive function.	Visuospatial memory errors reduced by 16%.	Positive
Ni et al. (22)	Prospective cohort study	Older adults (55–75 years) with overweight/ obesity and metabolic syndrome	747	6-year follow-up	Nut consumption	Global cognitive function, attention	Neuropsychological test battery	Consuming 3–7 servings of nuts/week showed slower declines in global cognitive function, interconnectedly associated with favorable gut microbiota.	4-year beta = 0.170, 6-year beta = 0.176.	Positive
Ni et al. (27),	Prospective cohort study	Adults 55–75 years with overweight/ obesity and metabolic syndrome	6,630	2-year follow-up	Nut consumption	Global, general, attention, and executive function	MMSE, CDT, VFT, TMT, DST	Frequent nut consumption was associated with a smaller decline in general cognitive performance over 2 years.	Nut intake >=7 servings/week had a beta z-score of 0.13.	Positive
Wu et al. (10)	Prospective cohort study	Middle-aged and older adults free from dementia at baseline	5,944	Median 6.0 years	Dietary fats (animal, vegetable, specific fatty acids)	Incident dementia	Langa-Weir classification strategy (TICS)	Higher vegetable fat and MUFA intake lowered dementia risk, while higher SFA intake increased risk. Replacing animal fat with vegetable fat lowered risk.	Vegetable fat HR = 0.69; MUFA HR = 0.63; SFA HR = 1.56.	Mixed (Depends on fat type)
Kien and Dumas (23)	Crossover RCT pilot	Older adults (65–75 years)	10	4-week feeding study	High vs Low Palmitic Acid to Oleic Acid ratio diets	Working memory	N-back task during fMRI	Lower Palmitic Acid intake increased activation in the brain's working memory network and showed a trend for improved working memory.	Working memory accuracy trend: *d*' = 1.62 vs 1.13 (*p* = 0.09).	Positive
Buchholz et al. (11)	RCT	Older adults with mild cognitive impairment due to Alzheimer's disease	38	12 weeks	Modified Atkins diet (MAD)	Memory, visuospatial memory, general cognition	MoCA, MMSE-2, CDR, HVLT-R, BVMT-R	MAD participants showed greater, albeit nonsignificant, improvement in memory.	Improvements were not statistically significant.	Nonsignificant positive trend
Norgren et al. (24)	Mixed regression models	Nondiabetic older adults at risk of dementia	676	2 years	Macronutrients (carbohydrate/fat ratio, protein, fiber, saturated/total fat)	Global cognition, memory, executive function, processing speed	Modified Neuropsychological test Battery (NTB)	APOE-genotype and insulin modulated the effect of dietary macronutrients on cognition, with APOE-e4 carriers showing different responses to carbohydrate/fat ratios.	Carbohydrate/fat ratio interaction with APOE-gradient beta = −0.040.	Mixed (Interaction effect)
Ting et al. (14)	Cross-sectional observational	Alzheimer's disease patients	592	Cross-sectional	Dietary patterns (frequencies of skimmed milk, pork, coffee/tea)	General cognition, functional domains	MMSE, Everyday cognition (E-Cog)	Higher frequencies of coffee/tea directly influenced cognitive outcomes, with gender differences in the influence of B12 and homocysteine.	Coffee/tea positively predicted MMSE (LASSO coef = 0.589).	Positive (for specific foods)
O'Donoghue et al. (15)	Pilot study	Healthy older Irish adults (65–85 years)	29	Cross-sectional	Dietary constituents vs EFSA dietary reference values	Spatial working memory, pattern recognition memory, visual learning, language	CANTAB	Cognitive performance was poorer in participants consuming excessive carbohydrates and PUFAs, and better with higher SFA/MUFA intake.	PRM accuracy lower for high carbohydrates (*r* = 0.60).	Mixed (Nutrient-dependent)
Cheatham et al. (25)	Double-blind, placebo-RCT	Adults aged 65–80 experiencing mild cognitive decline	131	6 months	Lyophilized wild blueberry powder	Speed of processing, spatial working memory, paired associates learning	CANTAB, MoCA	Wild blueberries improved speed of processing in participants with mild cognitive decline.	Powered to detect a medium effect size (Cohen's *f* = 0.25).	Positive
Amone et al. (26)	RCT	Healthy older adults	120	84 days (12 weeks)	Standardized Grape (Vitis vinifera L.) Extract	Immediate memory, visuospatial/constructional abilities, language, and attention	MMSE, RBANS, ENB-2	Grape extract significantly improved immediate memory, visuospatial/constructional abilities, language, and attention compared to placebo.	Immediate memory improved 16.0 and 12.8% at D28 and D84; language improved 24.2%.	Positive

### 3.1 Mediterranean diet approaches

The Mediterranean diet is consistently associated with superior global cognition and a deceleration of cognitive decline in older populations. A learning program focusing on the Mediterranean diet significantly improved dietary behaviors and global cognitive function among community-dwelling older adults (16). Similarly, short-term interventions have demonstrated that adherence to a Mediterranean diet, even without additional physical activity, effectively improves general cognition and verbal memory over a 24-week period (17). Over a longer term, a 3-year intervention utilizing an energy-restricted Mediterranean diet prevented age-related cognitive decline, with higher adherence directly linking to greater memory improvement (18). The clinical benefits of this dietary pattern also interact significantly with genetic risk factors. For instance, low adherence to the Mediterranean diet significantly increased the incidence of Alzheimer's disease specifically in individuals with low genetic risk (19). Furthermore, cognitive responses to the Mediterranean diet are modulated by an individual's sex and APOE genotype, with men experiencing greater memory improvements than women through the optimization of circulating endocannabinoid ratios (13). Specifically, optimizing these lipid mediators yields interconnected metabolic and cognitive benefits, such as improving insulin sensitivity and facilitating memory consolidation. While men experience these specific improvements, the cognitive benefits of APOE ε4 carriers vary significantly depending on the intervention duration. During the first year of intervention, APOE ε4 carriers exhibit positive cognitive changes, with effect sizes that are generally greater than those of noncarriers. However, over a longer-term 3-year period, these cognitive improvements appear less favorable for APOE ε4 carriers. Specifically, after 3 years, noncarriers continue to improve in global cognition and executive functioning, whereas APOE ε4 carriers show no significant changes on average. Individuals with the APOE ε4 allele show unique cognitive responses to specific carbohydrate-to-fat ratios, potentially requiring more personalized, macronutrient-adjusted approaches to optimize long-term cognitive outcomes (13). For a breakdown of each diet and the associated included articles, please see Table 2.

### 3.2 Vegetarian, plant-based diets, and nut consumption

Adopting nutrient-dense, plant-rich dietary patterns offers profound physiological benefits that protect cognitive health. Following a plant-rich diet is associated with a slower pace of epigenetic aging and lower inflammatory indicators compared to a standard American diet (20). Specifically, replacing animal fats and saturated fatty acids with vegetable fats, which are fundamental to plant-based diets, is strongly linked to a lower risk of developing dementia (10). Nut consumption is a key component of these diets and provides significant neuroprotective effects. A randomized crossover trial demonstrated that longer-term mixed nut consumption beneficially affected brain vascular function and improved both visuospatial and verbal memory in older adults (21). Additionally, regular nut consumption has been shown to alter the gut microbiota by increasing beneficial bacteria, which correlates with a slower decline in global cognitive function (22).

### 3.3 Macronutrient ratios, dietary fats, and ketogenic approaches

Modifying specific macronutrient ratios and fat types can yield significant cognitive benefits, aligning with principles often found in Nordic or low-carbohydrate dietary patterns. Research indicates that lowering the ratio of palmitic acid to oleic acid in the diet decreases systemic inflammation and increases activation in the brain's working memory network, showing a positive trend for improved working memory accuracy (23). The estimated effects of these dietary macronutrients on cognitive performance are further modulated by insulin status and APOE genotype, meaning that individuals with the APOE-e4 allele show unique cognitive responses to specific carbohydrate-to-fat ratios and polyunsaturated fats (24). Exploring more restrictive low-carbohydrate models, a randomized feasibility trial of the modified Atkins diet, a ketogenic approach, demonstrated that participants showed greater, albeit nonsignificant, improvements in memory along with increased circulating acetoacetate (11).

### 3.4 Okinawan diet components, micronutrients, and specific extracts

The Okinawan diet and its associated micronutrients and plant extracts offer targeted, domain-based cognitive benefits. The status of specific micronutrients in the blood is closely tied to cognitive outcomes and inflammation. For example, deficiencies in magnesium and vitamin B12, along with high zinc levels, correlate with increased systemic inflammation and lower cognitive task accuracy (15). Furthermore, an analysis of dietary patterns in Alzheimer's disease patients revealed that higher frequencies of coffee and tea consumption directly influenced cognitive outcomes, while the predictive value of B12 and homocysteine levels on cognition differed significantly between men and women (14). Targeted plant extracts prominent in whole-food diets also showed specific benefits. A 6-month intervention with wild blueberry powder improved the speed of processing in older adults experiencing mild cognitive decline (25). Similarly, a standardized grape extract intervention yielded significant improvements in immediate memory, visuospatial and constructional abilities, language, and attention when compared to a placebo (26).

## 4 Discussion

### 4.1 Summary of evidence

The findings of this systematic review demonstrate that high adherence to comprehensive, whole-food dietary patterns, specifically the Mediterranean, Nordic, Okinawan, and plant-based approaches, consistently mitigates cognitive decline and reduces the risk of Alzheimer's disease and related dementias in older adults (18). Because direct comparative evidence among these distinct dietary patterns remains limited, this review does not attempt to determine a single superior diet. Instead, our findings highlight that the Mediterranean, Nordic, Okinawan, and plant-based diets share essential, neuroprotective features, including high antioxidant density and favorable fatty acid profiles. Rather than relying on isolated nutrient supplementation, these dietary models leverage the complex synergy of the food matrix to induce beneficial physiological changes. For example, higher long-term consumption of nuts as part of a whole-food diet is directly associated with preserved cognitive performance and delayed cognitive decline (27). While the Mediterranean and Nordic diets utilize entirely different regional foods, both sustainable dietary models share similar physiological mechanisms for reducing systemic inflammation and promoting long-term cardiovascular health (28). Across the 16 included studies, despite utilizing different regional foods, these whole-food interventions successfully modulated the gut-brain axis, reduced systemic inflammation, optimized circulating endocannabinoids, and improved long-term cardiovascular health, translating to measurable protections in global cognition, memory, and executive function. Ultimately, the most effective dietary pattern may not be universally superior, but rather the one most appropriately tailored to an individual's unique metabolic status and genetic risk factors.

### 4.2 Clinical significance and current public health initiatives

Implementing these dietary patterns in long-term care settings presents a highly effective strategy to combat the clinical vulnerabilities unique to institutionalized older adults. Residents frequently suffer from accelerated physical and cognitive frailty, a decline that is closely tied to malnutrition, systemic inflammation, and sarcopenia (1, 15). Adhering to healthy, nutrient-dense dietary patterns has been shown to successfully prevent cognitive decline even in older adults already diagnosed with sarcopenia (29). Dietary interventions must account for the multidimensional nature of food insecurity in aging populations. Beyond simple economic resource constraints, older adults frequently experience food insecurity driven by physical functioning limitations, such as difficulty preparing meals or using utensils, which severely restricts their intake of healthy foods and is robustly linked to depression and poor self-reported health (30). By prioritizing nutrient-dense, plant-rich diets in institutional care, providers can better support residents' physical limitations while simultaneously promoting a slower pace of epigenetic aging and lowering overall inflammatory indicators (20).

Currently, several federal and state-level initiatives attempt to address these nutritional deficits in aging populations. Programs such as the Supplemental Nutrition Assistance Program (SNAP) (31), the Commodity Supplemental Food Program (CSFP) (32), the Senior Farmers Market Nutrition Program (SFMNP) (33), and Medicare Advantage Part C food benefits operate primarily by providing financial subsidies or direct commodity distributions to alleviate resource-constrained food insecurity (34). While these programs successfully increase overall caloric access and reduce economic barriers to food, they are generally designed to address basic hunger rather than the specific physiological demands of cognitive aging. To bridge this gap, structured nutritional education and behavioral support programs can be highly effective. Interactive, game-based Mediterranean diet learning programs have been shown to significantly improve both dietary behavior and global cognitive function in older adults (16). Similarly, short-term structured interventions promoting the Mediterranean diet alongside active behavioral support yield measurable improvements in general cognition and verbal memory (17).

### 4.3 Future research

A focus on longitudinal interventions that promote plant-based, vegetable-fat-rich, and nutrient dense eating patterns can help determine their sustained influence on inflammation, vascular health, and metabolic control. For example, further studies are needed to definitively map how altering specific macronutrient ratios, such as lowering the palmitic acid to oleic acid ratio, can decrease pro-inflammatory cytokines and increase activation in the brain's working memory network (23). Similarly, future research should explore how these dietary patterns modulate the endocannabinoid system to yield interconnected metabolic and cognitive benefits. Specifically, investigations could evaluate how diet-induced reductions in 2-arachidonoylglycerol improve insulin sensitivity and lower triglycerides to protect against synapse impairment, and how increasing the oleoylethanolamide to anandamide ratio supports memory consolidation by improving glucose homeostasis (13). To sustainably address nutritional inadequacy on a global scale, public health policies must expand the biofortification of staple crops to combat hidden hunger, including iron, zinc, and vitamin A deficiencies, which remain severe contributors to anemia and cognitive impairment (35, 36). The success of these population-level initiatives will rely heavily on the rigorous monitoring of biofortification program performance to ensure these essential micronutrients reliably reach vulnerable demographics (37).

### 4.4 Future personalized nutrition strategies

There is an urgent need to explore how comprehensive dietary patterns interact with non-modifiable biological factors, such as genetic risk, insulin status, and specific nutrient exposures (19). For instance, cognitive responses to specific macronutrient ratios and circulating endocannabinoids differ significantly based on an individual's sex and APOE genotype (13, 24). Although a quantitative meta-analysis and independent subgroup analyses were not performed in this review due to study heterogeneity, the modifying effects of sex and APOE genotype are strongly supported by the qualitative synthesis of the included primary literature (13). Specifically, primary studies were utilized that independently stratified their data, demonstrating significant biological interactions that support the need for personalized nutrition. Additionally, there are clear gender differences in how the folate, vitamin B12, and homocysteine axis influences cognitive outcomes in patients with Alzheimer's disease (14). Personalized strategies must also consider the targeted use of specific plant extracts, as wild blueberry powder has been shown to improve processing speed (25), and standardized grape extracts can significantly enhance immediate memory, language, and attention (26). Future studies utilizing standardized dietary measures alongside advanced biomarker-based methods, such as profiling the gut microbiome (22) and utilizing advanced epigenetic clocks like DunedinPACE (20), will help clarify causal mechanisms. Understanding these complex interactions will support the development of personalized nutrition strategies, allowing clinicians to tailor interventions that address the diverse biological factors influencing cognitive aging (5).

### 4.5 Strengths and limitations

A primary strength of this systematic review is its comprehensive evaluation of multiple distinct, whole-food, dietary patterns, moving beyond single-nutrient analyses to reflect real-world nutritional synergy. Additionally, the inclusion of rigorously assessed randomized controlled trials and large-scale prospective cohort studies strengthens the reliability of the clinical outcomes reported. However, this review is not without limitations. The inherent heterogeneity in dietary assessment tools, such as variations in food-frequency questionnaires across different studies, may introduce recall bias. Furthermore, while the included studies effectively highlight associations between diet and cognitive preservation, the reliance on observational data in several of the cohorts limits the ability to establish strict causality. Although the randomized controlled trials in our synthesis provide insight into short-term clinical efficacy, the long-term observational findings should be interpreted as strong associations rather than definitive causation. Future research must prioritize long-term, tightly controlled randomized clinical trials to definitively map the physiological pathways linking whole-food diets to dementia prevention.

## 5 Conclusion

This systematic review of 16 peer-reviewed articles demonstrates that comprehensive whole-food dietary patterns provide a more effective framework for preserving cognitive function than isolated nutrient interventions. Adherence to Mediterranean, Nordic, Okinawan, and healthy plant-based diets is consistently associated with improved memory, faster processing speeds, and a reduced risk of Alzheimer's disease and related dementias. These benefits are driven by a variety of biological mechanisms, including reduced systemic inflammation, improved vascular integrity, and favorable shifts in the gut microbiome. As observed in global longevity hotspots, these specific nutritional strategies converge to maintain metabolic flexibility and support healthy aging trajectories, proving that diet exerts a decisive influence on both lifespan and health span. For long-term care leaders and healthcare providers, these findings offer evidence-based strategies to improve resident outcomes through proactive nutritional planning. Moving forward, the integration of nutrient-dense dietary patterns and the strict reduction of ultra-processed foods must become a priority in clinical guidelines to support cognitive resilience and enhance the overall quality of life for the aging global population. While these findings highlight robust associations between whole-food dietary patterns and cognitive preservation, the observational nature of several included studies limits the ability to establish strict causality. Consequently, future long-term randomized clinical trials are necessary to confirm these neuroprotective effects and definitively map the underlying physiological pathways.
